# Inactivation kinetics of horseradish peroxidase (HRP) by hydrogen peroxide

**DOI:** 10.1038/s41598-023-39687-1

**Published:** 2023-08-17

**Authors:** Diego Morales-Urrea, Alex López-Córdoba, Edgardo M. Contreras

**Affiliations:** 1 Grupo de Investigación en Bioeconomía y Sostenibilidad Agroalimentaria, Escuela de Administración de Empresas Agropecuarias, Facultad Seccional Duitama, Universidad Pedagógica y Tecnológica de Colombia, Duitama, Colombia; 2 Instituto de Investigaciones en Ciencia y Tecnología de Materiales (INTEMA), CCT - Mar del Plata. CONICET, Mar del Plata, Argentina

**Keywords:** Biochemistry, Biotechnology

## Abstract

In recent years, the peroxidase enzymes have generated wide interest in several industrial processes, such as wastewater treatments, food processing, pharmaceuticals, and the production of fine chemicals. However, the low stability of the peroxidases in the presence of hydrogen peroxide (H_2_O_2_) has limited its commercial use. In the present work, the effect of H_2_O_2_ on the inactivation of horseradish peroxidase (HRP) was evaluated. Three states of HRP (E_0_, E_2_, and E_3_) were identified. While in the absence of H_2_O_2_, the resting state E_0_ was observed, in the presence of low and high concentrations of H_2_O_2_, E_2,_ and E_3_ were found, respectively. The results showed that HRP catalyzed the H_2_O_2_ decomposition, forming the species E_x_, which was catalytically inactive. Results suggest that this loss of enzymatic activity is an intrinsic characteristic of the studied HRP. A model from a modified version of the Dunford mechanism of peroxidases was developed, which was validated against experimental data and findings reported by the literature.

## Introduction

In recent years, biocatalysis has become a standard technology in several industrial processes, with hydrolases and redox enzymes as the most used ones^1^. Among redox enzymes, several applications of heme–iron oxidases, such as oxygenases and peroxidases have been reported. Besides the classical use of heme–iron oxidases to decolorize azo-dyes containing wastewaters^2–4^, and the polymerization of phenolic compounds^5–7^, these enzymes have been employed in the biosynthesis of several pharmaceuticals, such as polyketide antibiotics, artemisinin, and paclitaxel^8^. In particular, peroxidases were used in the lignin degradation for biofuel production^9^, in the asymmetric oxidations of amino acids^10^, and other syntheses of fine chemicals^11^, to prepare ‘antibody-enzyme’ or ‘antibody-enzyme conjugates for ELISA kits^12^, and in the enzymatic grafting of functional molecules^13^.

Up to the present, commercial uses of peroxidases have been limited by the low stability of HRP in the presence of hydrogen peroxide. In this sense, a deeper knowledge of peroxidase deactivation kinetics is necessary to develop more robust biocatalytic processes. For this reason, the objective of this work was to study the deactivation kinetics of a commercial HRP by hydrogen peroxide under a wide range of experimental conditions. A model based on a modified version of the Dunford mechanism of peroxidases was developed, validating the experimental data and findings reported in the literature.

## Theory

Extensive studies^14–16^ have demonstrated that the catalytic cycle of peroxidases involves several enzymatic species. These models are modifications or extensions of the Dunford mechanism^17^. According to Dunford and Stillman^17^, the catalytic cycle starts with the oxidation of the ground state of the enzyme (E_0_) by a peroxide to form the compound I (E_1_):

R1 $${E}_{0}+{H}_{2}{O}_{2}\stackrel{{k}_{o}}{\to }{E}_{1}+{H}_{2}O.$$

According to several authors, this reaction is very fast, being the second-order constant k_0_ about 1.5 × 10^7^ M^−1^ s^−1^^18,19^. This value was used for all further calculations.

Species E_1_ is two-electron equivalents above E_0_. Under the presence of an external reducing substrate (S), a sequence of two one-electron transfer occurs to restore E_0_ as follows:

R2 $${E}_{1}+S\stackrel{{k}_{1s}}{\to }{E}_{2}+{S}^{*}\to \to OP,$$

R3 $${E}_{2}+S\stackrel{{k}_{2s}}{\to }{E}_{0}+{S}^{*}\to \to OP.$$

The first one-electron reaction (R2) produces the compound II (E_2_) and a radical of the reducing compound (S*). Then, a second substrate molecule reacts with E_2_ to restore E_0_, producing another substrate radical (R3). As a general rule, the presence of these radicals results in a complex mixture of oxidation products (OP) which includes dimers, trimers, and oligomers of the parent substrate^2,14^ that cannot be further oxidized by hydrogen peroxide under the tested conditions.

Several authors report that the presence of organic impurities in the enzyme extract can serve as electron donors. Moreover, amino acids from the protein backbone and also the porphyrin ring itself can act as reducing substrates^20–22^. Accordingly, even the purest enzyme preparation always contains an unknown amount of these internal reducing substrates (${S}_{i}$) which can also be oxidized via reactions analogous to (R2) and (R3):

R2i $${E}_{1}+{S}_{i}\stackrel{{k}_{1si}}{\to }{E}_{2}+{S}_{i}^{*},$$

R3i $${E}_{2}+{S}_{i}\stackrel{{k}_{2si}}{\to }{E}_{0}+{S}_{i}^{*},$$

where the subindex i indicates that the rate constants ${k}_{1si}$, and ${k}_{2si}$ correspond to the oxidation of the internal substrate ${S}_{i}$ by ${E}_{1}$, and ${E}_{2}$, respectively.

Peroxidases can also catalyze the decomposition of hydrogen peroxide to oxygen and water (e.g., the catalasic cycle) as follows^18,21,23^:

R4 $${E}_{1}+{H}_{2}{O}_{2}\stackrel{{k}_{1p}}{\to }{E}_{2}+{HO}_{2}^{*},$$

R5 $${E}_{2}+{H}_{2}{O}_{2}\stackrel{{k}_{2p}}{\to }{E}_{3}.$$

Then, compound III (E_3_) slowly decays to E_0_, releasing an hydroperoxyl radical:

R6 $${E}_{3}\stackrel{{k}_{3}}{\to }{E}_{0}+{HO}_{2}^{*}.$$

Several authors also report the formation of an inactive enzyme species (E_X_) during the decay of E_3_. This new species is characterized by a decrease of the Soret band due to the loss of Fe atom (heme bleaching) and the presence of a new absorption band at 670 nm^22,24,25^:

R7 $${E}_{3}\stackrel{{k}_{d}}{\to }{E}_{X},$$

where E_x_ represents the enzyme decay product.

Reactions (R2) and (R4) are analogous in the sense that in both cases, a reducing compound (S in (R2), or ${H}_{2}{O}_{2}$ in (R4)) reduces E_1_ to E_2_, releasing a radical of the corresponding compound (${S}^{*}$ or ${HO}_{2}^{*}$). Similarly, (R3) and (R6) restore E_0_ from E_2_, releasing the corresponding radicals in both cases. Then, hydroperoxyl radicals (${HO}_{2}^{*}$) regenerate part of the consumed hydrogen peroxide, releasing molecular oxygen as follows^21^:

R8 $${HO}_{2}^{*}+{HO}_{2}^{*}\stackrel{{k}_{obs}}{\to }{H}_{2}{O}_{2}+{O}_{2},$$

where ${k}_{obs}$ is a function of pH. Taking into account that in the present work all assays were performed at pH = 9, according to Klassen and Ross^26^ k_obs_ = 5050 M^−1^ s^−1^. This value was used for all further calculations. For more details, see Supplementary Data, Item 1.

## Materials and methods

### Chemicals and reagents

Horseradish peroxidase (HRP) (Type I, RZ = 1.1) was from Sigma-Aldrich. HRP was supplied as a lyophilized powder and it was used without further purification. According to the manufacturer, the specific activity was 146 units/mg of powder (one unit corresponds to the amount of enzyme that forms 1 mg of purpurogallin from pyrogallol in 20 s at pH 6 and 20 °C). Hydrogen peroxide (H_2_O_2_) (30 wt%) was from Sigma-Aldrich. Analytical grade (> 98%) Orange II (OII) sodium salt (CAS # 633-96-5) was used as the external reducing substrate for the measurement of the enzyme activity. OII was used without further purification. All other salts used in this work were reagent grade from Anedra (San Fernando, Argentina).

### UV–Vis spectra of HRP species

In a first set of experiments, UV–Vis spectra corresponding to the different enzymatic species were obtained as follows. Firstly, a stock solution of the enzyme was prepared, mixing 50 mg of HRP into 100 mL of a phosphate buffer (PB) 100 mM at pH 9. Then, 3 mL of this solution was poured into a quartz cuvette and the UV–Vis spectrum corresponding to E_0_ was recorded against the PB as the cell blank. Then, several additions of 20 µL of H_2_O_2_ (1 mM) were performed until the UV–Vis spectrum of the reaction mixture became stable for, at least, 10 min. To obtain the UV–Vis spectrum corresponding to compound III (E_3_), 3 mL of the HRP stock solution and 20 µL of H_2_O_2_ (260 mM) were mixed^22^. Finally, 2.5 mL of an HRP solution containing 200 to 500 mg/L of the lyophilized enzyme in PB (100 mM, pH 9) was mixed with 50 µL of H_2_O_2_ (260 mM, pH 9). After 6 h of reaction time, the UV–Vis spectrum corresponding to the inactive species of the enzyme (E_X_) was obtained^24,27^.

### HRP inactivation experiments

To evaluate the inactivation of HRP by H_2_O_2_, 2.5–12.5 mg of the HRP lyophilized powder was dissolved in 25 mL of phosphate buffer (PB) 100 mM, pH 9. Prior to the addition of H_2_O_2_, the UV–Vis spectrum of the reaction mixture was recorded. Then, the reaction was started by the addition of 15 µL of H_2_O_2_ (9.8 M). At predefined time intervals, samples were taken to measure the H_2_O_2_ concentration, enzyme activity, and UV–Vis spectra of the reaction mixture. All inactivation experiments were performed at room temperature. The determinations of the H_2_O_2_ concentration and enzyme activity were performed in triplicate.

### Analysis of UV/Vis spectra

The analysis of the obtained UV/Vis spectra was based on the multicomponent Beer’s law^28^. Thus, it was assumed that a given UV/Vis spectrum (${A}_{\lambda }$) can be represented as the sum of individual spectra corresponding to each enzyme species in the reaction mixture:

1 $${A}_{\lambda }={\varepsilon }_{0,\lambda }\left[{E}_{0}\right]+{\varepsilon }_{2,\lambda }\left[{E}_{2}\right]+{\varepsilon }_{3,\lambda }\left[{E}_{3}\right]+{\varepsilon }_{x,\lambda }\left[{E}_{X}\right],$$

where ${\varepsilon }_{i,\lambda }$ and $\left[{E}_{i}\right]$ represent the spectrum and the concentration corresponding to the species i, respectively. The spectra corresponding to the individual species (${\varepsilon }_{i,\lambda }$) were obtained as it was commented in Section “UV-Vis spectra of HRP species” Then, by fitting Eq. (1) to the experimental absorption spectrum, the concentration corresponding to each enzyme species was obtained. The fitting procedure was implemented in SigmaPlot 10.0. For details, see Supplementary Data, Item 2.

### Enzyme activity assay

The peroxidatic activity of the studied HRP was measured according to Morales et al.^2^. Briefly, 2 mL of phosphate buffer (PB) 100 mM, pH 9, and 200 µL of the solution containing the enzyme were mixed. Then, 100 µL of OII (2 mM) was added as the reducing substrate (electron donor). Finally, the reaction started with the addition of 100 µL of H_2_O_2_ (9.8 mM). The consumption of OII was monitored at 485 nm. Results were expressed as initial decolorization rate (VD, a.u./s). All determinations were performed in triplicate.

### Analytical methods

UV–Vis absorption spectra were recorded at room temperature (~ 20 °C) in quartz cuvettes (1 cm) on a Shimadzu UV-1800 spectrophotometer using a spectral bandwidth of 1 nm and a scan speed of 1200 nm/min. The enzyme concentration was evaluated by measuring the absorbance at 403 nm using ε_E0-403_ = 103 mM^−1^ cm^−1^^29^. H_2_O_2_ concentration was measured by the Trinder method^30^. The determinations of Trinder method were performed in triplicate.

### Dynamical simulations and fitting procedure

All dynamical simulations and fittings were performed using the software GEPASI 3.30^31^. GEPASI integrates the systems of differential equations with the routine LSODA (Livermore Solver of Ordinary Differential Equations). LSODA algorithm switches the integration method between the Adams integration method with variable step size and variable order up to 12th order within nonstiff regions and the Gear (or BDF) method with variable step size and variable order up to 5th order for stiff regions. With regard to the fitting procedure, Multistart Optimization algorithm with Levenberge Marquardt local optimization was selected. Multistart is a hybrid stochastic-deterministic optimization method. Rather than run a simple local optimization, Multistart runs several of them. The first start takes for an initial guess the parameter values entered by the user. Then, initial guesses for the subsequent starts are generated randomly within specific boundaries that the user can set. The local optimizer used is the Levenberge Marquardt method, which has proved the most efficient gradient optimizer available in GEPASI^32^. The program developed in the GEPASI language is available at request.

## Results and discussion

### UV–Vis spectra corresponding to the enzyme species

**Figure 1:**
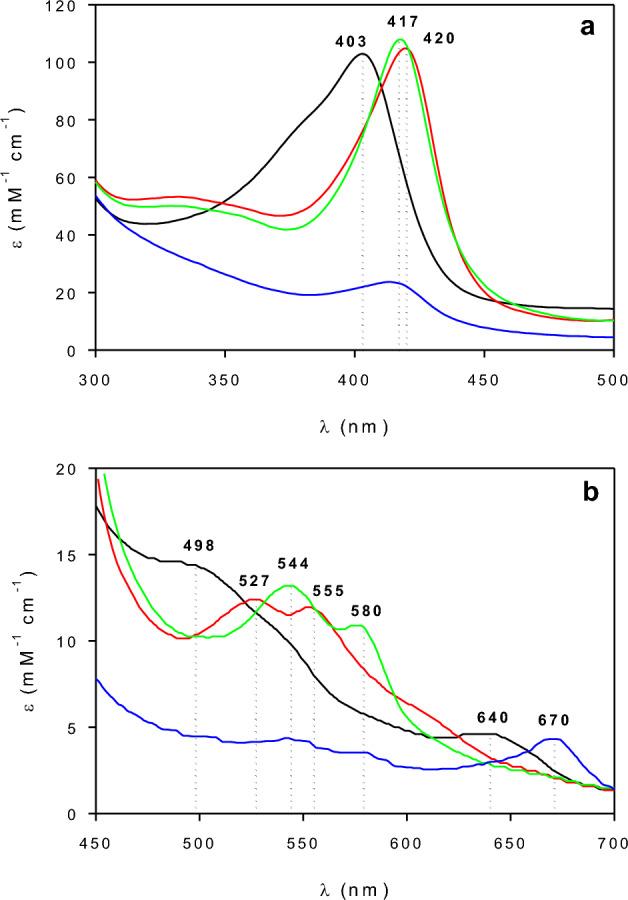
(**a**) Soret band, and (**b**) visible absorption spectra corresponding to the species E_0_ (black), E_2_ (red), E_3_ (green), and E_X_ (blue). Dotted lines indicate the characteristic absorption bands corresponding to each species.

Figure 1 shows the UV–Vis spectrum corresponding to the resting state of HRP (E_0_). Besides, the position corresponding to the maximum of the Soret band at 403 nm, the other two bands at 498 and 640 nm can be observed. The presence of these bands in the UV–Vis spectrum of E_0_ was also reported by other authors^18,29,33^.

UV–Vis spectrum corresponding to E_2_ was obtained by adding several pulses of H_2_O_2_ to the native enzyme. During the first additions of H_2_O_2_, observed spectra were unstable and after a few seconds, the spectrum corresponding to E_0_ was obtained. However, after several additions of H_2_O_2_, the spectrum corresponding to E_2_ became stable for, at least, 10 min. Figure 1 shows that the maximum of the Soret band corresponding to E_2_ shifted to 420 nm. Also, two characteristic bands corresponding to E_2_ at 527 and 555 nm were also noticeable. The reported extinction coefficient of E_2_ at 420 nm (ε_E2−420_) is 105 mM^−1^ cm^−1^^29^. Accordingly, this value was used to scale the spectrum corresponding to E_2_ (Fig. 1).

To obtain the UV–Vis spectrum corresponding to E_3_, the enzyme was mixed with an excess of H_2_O_2_^22^. It must be noted that in this case, it was necessary more than 200 times the amount of hydrogen peroxide required to obtain E_2_. Figure 1 shows that the maximum of the Soret band shifted from 403 to 417 nm. Moreover, two other new bands appeared at 544 and 580 nm. This behavior was also reported by several authors^29,33^ using different HRP isoenzymes. According to Ortiz de Montellano^29^, the extinction coefficient corresponding to E_3_ at 417 nm (ε_E3−417_) is 108 mM^−1^ cm^−1^. Hence, the spectrum corresponding to E_3_ was scaled using this extinction coefficient (Fig. 1).

The spectrum corresponding to E_3_ (Fig. 1) slowly changes as a function of time. According to several authors^22,24,27^ this new UV–Vis spectrum corresponded to the inactive form of the enzyme (E_X_). Figure 1 shows that after 6 h, the Soret band was almost absent in the UV–Vis spectrum of the reaction mixture. Additionally, this spectrum contained a new band at 670 nm due to the presence of biliverdin, an enzyme degradation product^22,27^. To scale the UV–Vis spectrum corresponding to E_X_, the extinction coefficient of biliverdin at 670 nm (ε_Ex−670_) of 4.3 mM^−1^ cm^−1^ was used^25^.

Under the tested conditions the formation of E_1_ could not be demonstrated. According to Ortiz de Montellano^29^, E_1_ exhibits an absorption band at 400 nm (Soret band), and the other three minor ones at 577, 622, and 651 nm. Although the maximum of the Soret band corresponding to E_1_ is close to E_0_, bands at 577, 622, and 651 nm are absent in all the obtained UV–Vis spectra (Fig. 1). In this sense, several works demonstrate the outstanding oxidizing capability of the E_1_, which is significantly higher than that E_2_^27,34,35^. Accordingly, it can be expected that under the presence of H_2_O_2_, the concentration of E_1_ would be much lower than E_2_.

### Interconversion of HRP species at low H_2_O_2_ concentration

**Figure 2:**
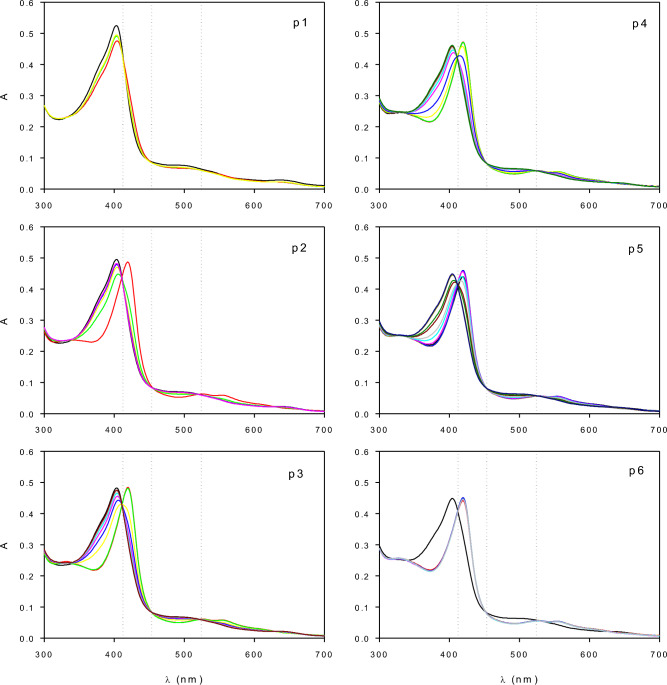
Change of the UV–Vis spectra of the reaction mixture as a function of time after the repeated additions of 20 µL of H_2_O_2_ (1 mM) to 3 mL of HRP (500 mg/L of lyophilized enzyme in PB 100 mM, pH 9). In each case, pi represents the number of the pulse of the oxidant.

Figure 2 shows the change of the UV–Vis spectrum corresponding to the reaction mixture as a function of time obtained during repeated additions of H_2_O_2_ to the tested HRP. In these assays, the ratio of hydrogen peroxide to the total enzyme was 1.3 mol/mol. After the first addition of H_2_O_2_, a fast change in the spectrum was observed (Fig. 2p1). Then, from pulses 2 to 5 (Fig. 2p2 to p5), obtained UV–Vis spectra exhibited at least three isosbestic points at 412, 453, and 524 nm, indicating a reversible transformation of the enzyme species. Rodriguez-Lopez et al.^36^, reported that UV–Vis spectra corresponding to E_0_ and E_2_ have an isosbestic point at 412 nm. Although those authors also reported an isosbestic at 395 nm corresponding to the transformation of E_1_ to E_2_, UV–Vis spectra depicted in Fig. 2 do not exhibit such isosbestic, confirming that the formation of E_1_ was negligible under the tested conditions.

**Figure 3:**
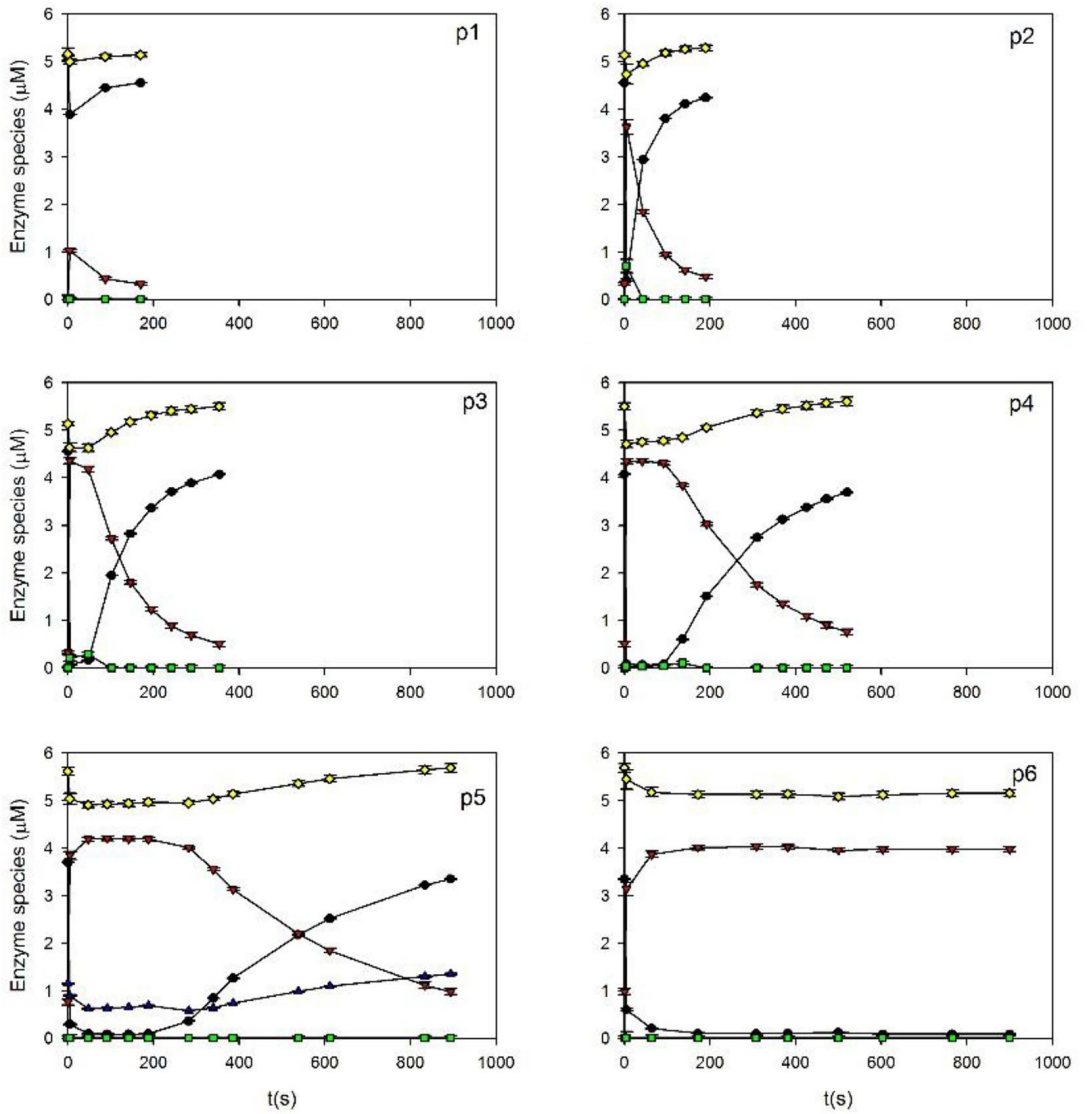
Enzyme species E_0_ (black), E_2_ (red), and E_3_ (green) as a function of time after the addition of successive pulses (p1 to p6) of H_2_O_2_ to HRP (500 mg/L) dissolved in phosphate buffer (100 mM, pH 9). Yellow symbols indicate the sum of all the species. Bars represent the standard deviation.

Based on the UV–Vis spectra corresponding to the identified enzyme species (Fig. 1), the concentration of each species was calculated as a function time from the spectra shown in Fig. 2. Details of these calculations can be found in Supplementary Material, Item 1. Figure 3 shows that after the first addition of H_2_O_2_ (Fig. 3p1), a fast transition from E_0_ to E_2_, and then back to E_0_ occurred. In this case, E_2_ only represented less than 20% of the total enzyme. However, after several additions of peroxide, this fraction gradually increased (Fig. 3p3 to p6). Moreover, from the comparison between Fig. 3p2 to p5, it can be noted that E_2_ decayed slower as a function of the added pulse of H_2_O_2_.

Reactions (R2) to (R5) show that the decay rate of E_2_ is a function of reducing substrate and hydrogen peroxide concentrations. According to reactions (R1) to (R3), successive additions of H_2_O_2_ to the same enzyme sample should cause a gradual consumption of the reducing substrate. Thus, the increasing stability of the UV–Vis spectrum corresponding to E_2_ after each addition of H_2_O_2_ (Fig. 3) suggested the consumption of an internal reducing substrate. Several authors report that the reduction of E_1_ to E_2_ even occurs in the absence of an external substrate. This effect was associated with both, the role of hydrogen peroxide as the reducing compound via (R4) and (R5), and also the presence of impurities in the enzyme extract (e.g. other proteins), amino acids from the protein backbone, and/or the porphyrin ring that can also act as internal reducing substrates^20–22^.

Results shown in Fig. 3 demonstrate that under the tested conditions E_0_ and E_2_ were the main enzyme species in the reaction mixture. Moreover, the sum of all the detected enzyme species was fairly constant as a function of time, suggesting that the decay of the enzyme via (R8) was negligible. For this reason, instead of the complete UV–Vis spectra, the conversion between these species can be monitored using a single wavelength. The advantage of using a single wavelength is that the reaction can be monitored on a continuous basis, improving the quality of the obtained data. To maximize the change of absorbance during the assay, the absorbance was measured at 422 nm (for more details, see Supplementary Material, Item 3).

**Figure 4:**
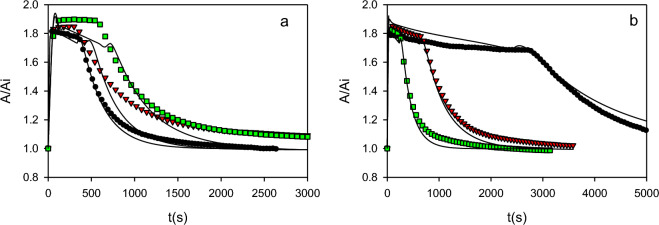
Typical examples of the effect of the initial concentration of H_2_O_2_ (**a**), and enzyme (**b**) on the change of absorbance at 422 nm. Experimental conditions in (**a**) HRP = 5.2 µM; H_2_O_2_ (µM) = 26 (black), 48 (red), 67 (green). Experimental conditions in (**b**) HRP (µM) = 1.7 (black), 3.4 (red), 5.2 (green); H_2_O_2_ = 26 µM. All assays were performed in PB (100 mM, pH 9) at room temperature. For comparison purposes, results were normalized with respect to the corresponding initial absorbance value (A_i_). Continuous lines indicate the proposed model using the coefficients shown in Table 1.

Figure 4 shows typical examples regarding the effect of the initial concentrations of H_2_O_2_ (Fig. 4a), and enzyme (Fig. 4b) on the change of the absorbance at 422 nm. Full results can be found in the Supplementary Material, Fig. A3. In all cases, when H_2_O_2_ was added to the reaction mixture, a fast increase in the absorbance due to the conversion of E_0_ to E_2_ was observed. In the cases when the initial H_2_O_2_ concentration was high enough the absorbance reached a plateau, indicating that all the enzyme was under the form E_2_. Figure 4 also shows that after a certain critical time, the absorbance started to decrease and eventually reached a value close to the initial one (e.g., A/A_i_ ≈ 1). In all cases, UV–Vis spectra corresponding to the final state corresponded to E_0_. Figure 4 shows that the above-mentioned critical time increased with the initial H_2_O_2_ concentration (Fig. 4a), and decreased with the initial enzyme concentration (Fig. 4b), which is strong evidence that the interconversion between E_2_ and E_0_ was due to the peroxide depletion.

**Table 1:** Kinetic coefficients corresponding to the proposed model ((R1) to (R9), and Eq. (2)).

Reaction	Coefficient	Units	Value	Reference
R1	${k}_{0}$	M^−1^ s^−1^	1.5 × 10^7^	^18,19^
R2	${k}_{1s}$	M^−1^ s^−1^	8400	This work
R3	${k}_{2s}$	M^−1^ s^−1^	7000	This work
R2i	${k}_{1si}$	M^−1^ s^−1^	1480	This work
R3i	${k}_{2si}$	M^−1^ s^−1^	240	This work
R4	${k}_{1P}$	M^−1^ s^−1^	4860	This work
R5	${k}_{2P}$	M^−1^ s^−1^	350	This work
R6	${k}_{3}$	s^−1^	0.03	This work
R7	${k}_{d}$	s^−1^	3.5 × 10^–4^	This work
R8	${k}_{obs}$	M^−1^ s^−1^	5050	^26^
R9	${k}_{Ex}$	M^−1^ s^−1^	90	This work
R2i/ R3i	${Y}_{{S}_{i}/E}$	mol/mol	8.59	This work

### HRP degradation by high H_2_O_2_ concentrations

**Figure 5:**
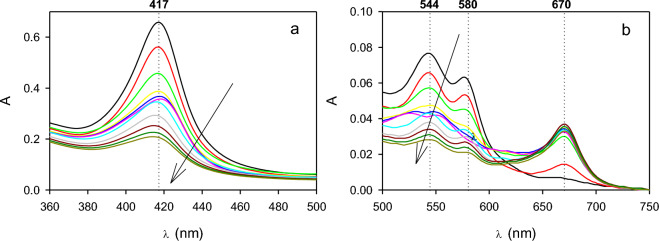
(**a**) Soret band, and (**b**) visible absorption spectra corresponding as a function of time. Initial conditions: HRP = 6 µM, H_2_O_2_ = 550 µM. Dotted lines indicate the characteristic absorption bands corresponding to E_3_ (417, 544, and 580 nm), and E_x_ (670 nm). Arrows indicate the evolution of time.

**Figure 6:**
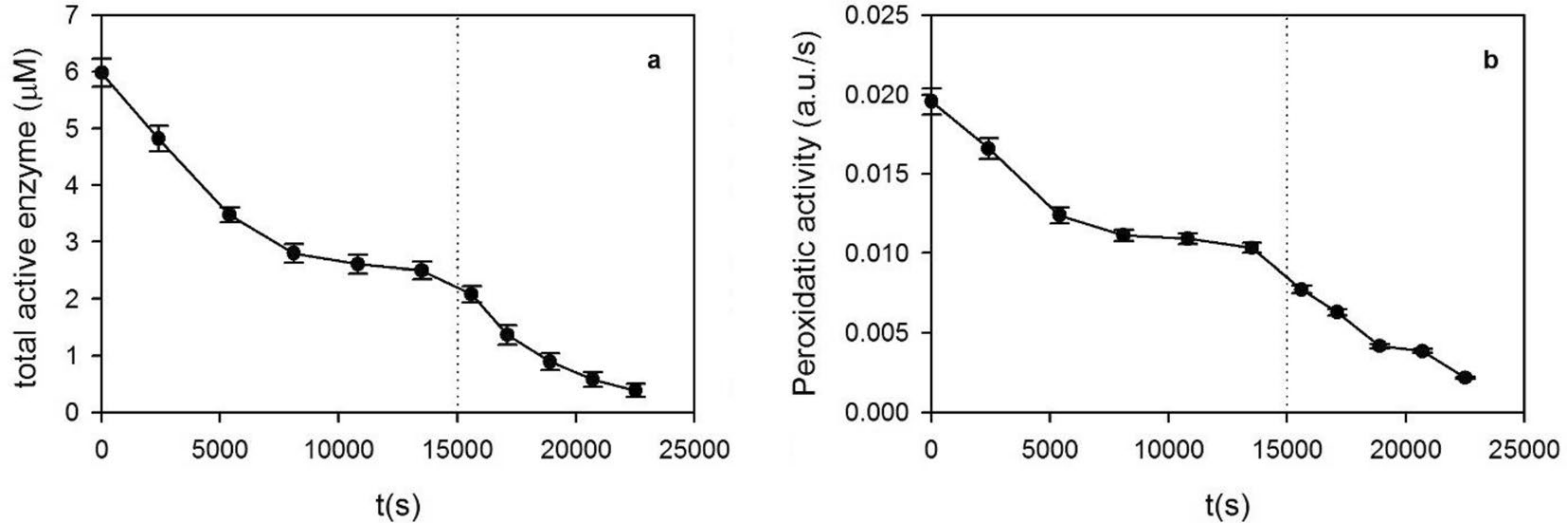
(**a**) Total active enzyme concentration (E_A_), and (**b**) peroxidatic activity as a function of time. Enzyme concentrations were obtained from the UV/Vis spectra depicted in Fig. 5. Bars represent the standard deviation. Dotted lines indicate the addition of a new pulse of H_2_O_2_.

To evaluate the inactivation of HRP by H_2_O_2_, the initial oxidant concentrations were increased concerning those used in the experiments described in the previous section. While in those assays the H_2_O_2_ concentration ranged between 20 and 53 µM (Fig. 4), in the experiments depicted in Figs. 5 and 6 the initial oxidant concentration was 550 µM. Figure 5 shows that peaks at 417, 544, and 580 nm, which correspond to E_3_ (see Fig. 1), decreased as a function of time. Besides, spectra depicted in Fig. 5b reveal the presence of a new absorption band at 670 nm, which corresponded to the formation of the enzyme oxidation product E_X_ (Fig. 1). In this sense, several authors report the formation of an inactive enzyme species under the excess of H_2_O_2_^24,27^. This inactive species is characterized by the heme bleaching (e.g., the decrease of the band at 417 nm, Fig. 5a) due to the loss of the Fe atom^22,37,38^. The analysis of the obtained spectra revealed that E_3_ and E_x_ were the main enzyme species, being negligible the concentration corresponding to E_0_ and E_2_. For this reason, E_3_ represented the main contribution to the total active enzyme concentration (e.g., the sum of E_0_, E_2_, and E_3_).

From the UV/Vis spectra depicted in Fig. 5, the total active enzyme concentration (E_A_) was obtained as a function of time. Figure 6a shows that E_A_ decreased from 6 to 2.5 µ M during the first 10,000 s and then remained constant due to the absence of H_2_O_2_. Then, at 15,000 s a new pulse of oxidant was added and the degradation of the enzyme continued. In parallel to UV/Vis spectra, the peroxidatic activity of the reaction mixture was also evaluated (full data are shown in the Supplementary Material, Fig. A4). Figure 6b shows that the peroxidatic activity (e.g., the oxidation of OII, the tested external electron donor) showed a similar trend as E_A_ (Fig. 6a), confirming the degradation of the enzyme.

**Figure 7:**
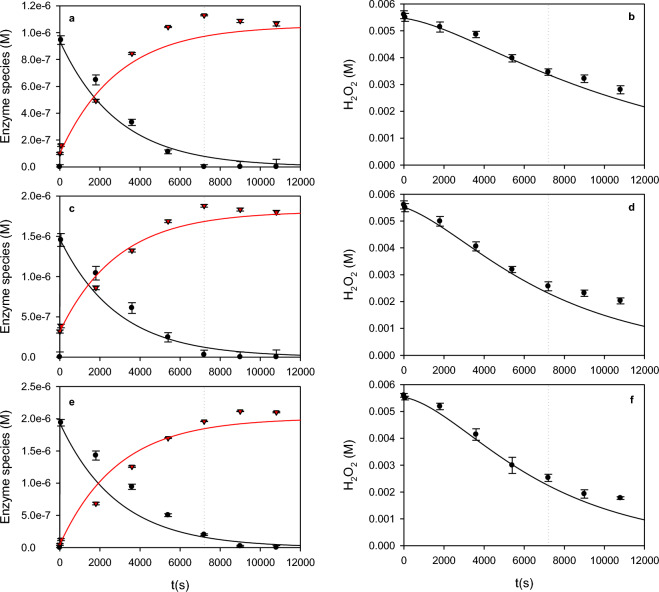
Degradation of E_3_ (black) and formation of E_x_ (red) (**a,c,e**) and the corresponding H_2_O_2_ consumption (**b,d,f**) as a function of time. Bars represent the standard deviation. Continuous lines indicate the proposed model using the coefficients shown in Table 1. Dotted lines represent the time at which the active enzyme concentration was less than 10% of the initial one.

**Figure 8:**
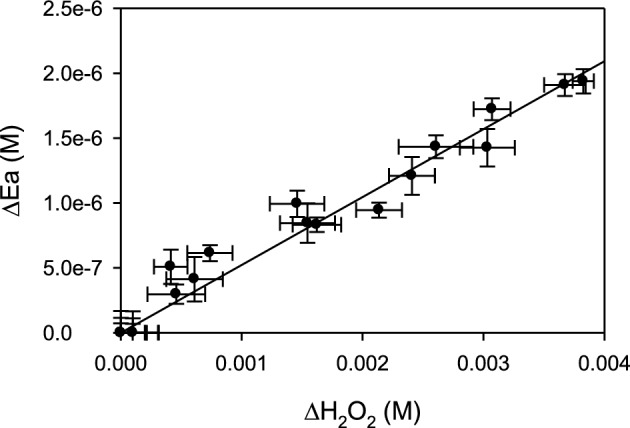
Degradation of HRP as a function of the consumed H_2_O_2_. Data were obtained from the results shown in Fig. 7. Bars represent the standard deviation.

Figure 7 shows the change of E_3_, E_x_, and H_2_O_2_ concentrations as a function of time obtained at three different initial HRP concentrations (UV/Vis spectra corresponding to these reaction mixtures can be found in the Supplementary Data, Fig. A5). In all cases, a decrease of E_3_ and the corresponding formation of E_x_ was obtained (Fig. 7a,c,e). Based on these data, the amount of degraded enzyme and H_2_O_2_ were obtained. Figure 8 shows that the enzyme degradation was proportional to the H_2_O_2_ decomposition, confirming that the enzyme degradation was an H_2_O_2_-dependent process. Besides, considering that control assays demonstrated that H_2_O_2_ decomposition was negligible in the absence of HRP, Fig. 7 shows that H_2_O_2_ was actively degraded by the presence of the enzyme. However, it is important to note that although the enzyme was almost completely degraded after about 7000 s (Fig. 7, dotted lines), the degradation of H_2_O_2_ still continued. This effect could be attributed to the presence of free Fe from heme bleaching^22^. In this sense, it is well known that H_2_O_2_ decomposition is favored by the presence of traces of free or complexed iron under alkaline conditions, such as those used in the present work^39–41^.

### Fitting the proposed HRP model

The basis of the model proposed in the present work consisted of reactions (R1) to (R8). Reactions (R1) to (R3) represent the Dunford mechanism of peroxidases for the oxidation of an external electron donor^17^. Reactions (R4) to (R6) correspond to the catalasic cycle of peroxidases^18,21,23^. Reaction (R7) represents the formation of the inactive enzyme species (E_X_) during the decay of E_3_^22,24,25^. Finally, (R8) is the recombination of hydroperoxyl radicals^21^. Then, this model was extended to take into account the oxidation of an internal electron donor (see Section “Interconversion of HRP species at low H_2_O_2_ concentration”) via reactions (R2i) and (R3i). For simplicity, it was assumed that the initial concentration of this internal substrate was proportional to the initial concentration of the native enzyme, being ${Y}_{{S}_{i}/E}$ (mol/mol) the proportionality constant. Finally, to take into account the consumption of H_2_O_2_ even after the inactivation of the enzyme (see Section “HRP degradation by high H_2_O_2_ concentrations”), a new H_2_O_2_ decomposition process was proposed assuming that the rate of this process (${R}_{{H}_{2}{O}_{2}}$) was proportional to both E_x_ and H_2_O_2_ concentrations:

R9 $${H}_{2}{O}_{2}\stackrel{{R}_{{H}_{2}{O}_{2}}}{\to }{H}_{2}O+\frac{1}{2}{O}_{2};\,\,\,\,\, {R}_{{H}_{2}{O}_{2}}={k}_{Ex}\left[{E}_{x}\right]\left[{H}_{2}{O}_{2}\right] .$$

In previous works, Morales et al.^2,7^, employed OII as the external substrate to evaluate the peroxidatic activity of HRP. Although OII had a strong absorbance peak at 485 nm (${\varepsilon }_{OII}=19500\, {cm}^{-1}{M}^{-1}$), those authors reported that OII oxidation products (OP) generated via (R2) and (R3) also absorbed significatively at this wavelength. Moreover, the molar attenuation coefficient corresponding to these OP depended on pH, being ${\varepsilon }_{OP}=900\, {cm}^{-1}{M}^{-1}$ at pH = 9^2^. Thus, when OII is employed as the external substrate to evaluate the peroxidatic activity of HRP (e.g., Fig. A4), the absorbance at 485 nm (A485) is the result of the presence of both OII and OP:

2 $$A485={\varepsilon }_{OII}\left[OII\right]+{\varepsilon }_{OP}\left[OP\right].$$

Equation (2) was used to calculate A485 as a function of the calculated concentrations of OII and OP (Fig. A4).

The model proposed in the present work ((R1) to (R9)) was fitted to the data depicted in Figs. 4, 6, 7 and Figs. A3, A4. Fitting results are shown in Table 1. Using these coefficients, simulations show a satisfactory representation of the available data by the proposed model (Figs. 4, 6, 7 and Figs. A3, A4). Moreover, the present model also predicts some findings reported by other authors. For example, several authors report a Haldane-type dependence of the peroxidatic activity as a function of the initial concentration of H_2_O_2_. Additionally, Haldane coefficients also depend on the initial external substrate concentration^2,14,42–44^. Besides, Morales et al.^2,7^ demonstrate that the amount of consumed H_2_O_2_ per mol of oxidized external substrate (OII) (Y_H2O2/Se_) increased from 0.5 mol/mol, which corresponds to the minimum value according to the Dunford mechanism ((R1) to (R3)), as a function of the initial H_2_O_2_ concentration. All these features can also be reproduced by means of the present model. Details of these calculations can be found in Supplementary Material, Item 4, and in Fig. A6.

**Figure 9:**
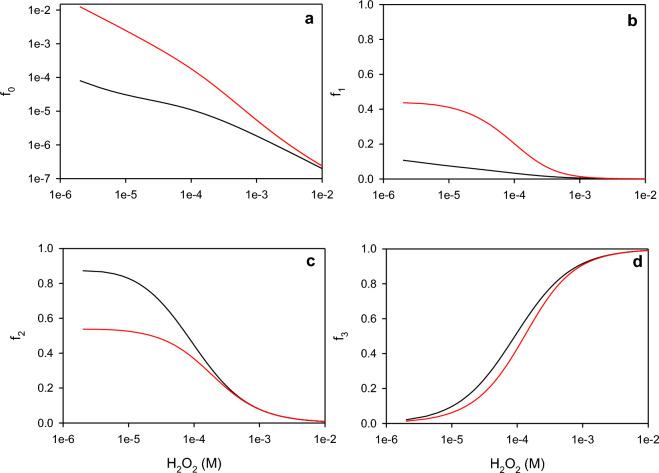
Effect of the hydrogen peroxide concentration on the fraction of each enzyme species under the absence (black lines) or the presence (red lines) of an external substrate (OII = 1 × 10^–4^ M). Calculations were performed using Eqs. (A26) to (A29) along with the coefficients shown in Table 1. For details, see the Supplementary Material, Item 5.

Several authors also reported that the addition of an external reducing compound, such as OII, can reduce the deactivation of peroxidases (R7) by preventing the formation of E_3_ (R5) through the competition between the substrate and H_2_O_2_ for E_2_ ((R3), (R3i))^18,22,45^. According to the present HRP model, the HRP deactivation rate is proportional to E_3_ (R7). Thus, to estimate the effect of the presence of an external substrate (e.g., OII) on the concentration of E_3_, a pseudo-steady state approximation was adopted. Details of these calculations can be found in the Supplementary Material, Item 5. Figure 9a demonstrates that regardless the H_2_O_2_ concentration, E_0_ represents less than 1% of the total enzyme. However, the presence of the external substrate increases the fraction corresponding to E_0_ from one to two orders of magnitude, depending on the H_2_O_2_ concentration. For intermediate H_2_O_2_ concentrations (e.g., 10^–5^ to 10^–4^ M), E_1_ and E_2_ are the main species (Fig. 9b,c). Within this H_2_O_2_ concentration range, the presence of the external substrate enhances the fraction corresponding to E_1_ (Fig. 9b) but prevents the formation of E_2_ (Fig. 9c). Moreover, because E_3_ is formed during the reaction of E_2_ with hydrogen peroxide via (R5), the presence of the external substrate also prevents the formation of E_3_ (Fig. 9d). Finally, for H_2_O_2_ concentrations higher than about 0.001 M, species E_3_ represents more than 90% of the enzyme, regardless the presence of the external substrate (Fig. 9d). It is important to note that as the reaction proceeds, the concentration of hydrogen peroxide and the external substrate decrease as a function of time. Thus, the presence of an external substrate not only diminishes the fraction of E_3_ at a given hydrogen peroxide concentration (Fig. 9d) but also enhances the consumption of the oxidant (Fig. A7), yielding lower H_2_O_2_ concentrations in comparison with the absence of the substrate. In fact, this is the main reason why the presence of an external substrate reduces the deactivation of the enzyme.

## Conclusions

In this work, the catalytic stability of an HRP was studied. Three states of HRP were identified: the basal state (E_0_) in the absence of H_2_O_2_, and species E_2_, and E_3_ in the presence of low concentrations or excess of H_2_O_2_, respectively. Besides, the decay of E_3_ generates a new E_x_ species, which is catalytically inactive. The proposed model adequately represents the inhibition of the enzymatic activity at high concentrations of H_2_O_2_. Although the presence of an external substrate (S_e_) prevents the formation of E_3_, and thereof, the degradation of the enzyme, the main protective effect of S_e_ can be attributed to the enhancement in the hydrogen peroxide consumption rate. Despite its status as a commercial enzyme, the obtained results demonstrated the catalasic activity of HRP. Consequently, further studies pertaining to the purification of this enzyme are deemed necessary.

### Supplementary Information

Supplementary Information.

## Data Availability

All data generated or analyzed during this study are included in this published article and its supplementary information file.
