# Supplementary material for: Inactivation kinetics of horseradish peroxidase (HRP) by hydrogen peroxide
Source: Sci Rep. 2023 Aug 17;13:13363. doi: 10.1038/s41598-023-39687-1 (PMC10435507; doi:10.1038/s41598-023-39687-1)
Supplement: Supplementary file 1 — Supplementary Information. [file 41598_2023_39687_MOESM1_ESM.pdf]

Inactivation kinetics of horseradish peroxidase (HRP) by hydrogen peroxide

- Supplementary Material -

Item 1. Reaction (R8) of the manuscript represents the recombination of hydroperoxyl radicals (HO_2^*) to produce hydrogen peroxide and molecular oxygen:

$$HO_2^* + HO_2^* \xrightarrow{k_{obs}} H_2O_2 + O_2 \qquad \text{(R8)}$$

where k_{obs} is an observed rate constant that depends on pH. According to this overall reaction, the oxygen production rate (v_{O2}) is:

$$v_{O2} = k_{obs}{C_T}^2 \qquad \text{(A1)}$$

where C_T is the total radicals concentration.

While in acid media this reaction proceeds via the direct combination of two hydroperoxyl radicals (eq. A2), at higher pH values superoxide radicals (O_2^{*-}) can also react with hydroperoxyl radicals (eq. A2):

$$HO_2^* + HO_2^* \xrightarrow{k_H} H_2O_2 + O_2 \qquad \text{(A2)}$$

$$HO_2^* + O_2^{*-}(+H_2O) \xrightarrow{k_{OH}} H_2O_2 + O_2 + OH^- \qquad \text{(A3)}$$

where $k_H = 6.64\,x\,10^5 M^{-1}s^{-1}$, and $k_{OH} = 7.58\,x\,10^7 M^{-1}s^{-1}$. Although hydroperoxyl and superoxide radicals can also react with hydrogen peroxide to give oxygen and hydroxyl radicals, respectively, these reactions are very slow and can be neglected (Klassen and Ross, 1997).

According to reactions A2 and A3, the oxygen production rate (v_{O2}) is

$$v_{O2} = k_H[HO_2^*]^2 + k_{OH}[HO_2^*][O_2^{*-}] \qquad \text{(A4)}$$

Besides, the acid-base equilibrium between hydroperoxyl (HO_2^*) and superoxide (O_2^{*-}) radicals is as follows:

$$HO_2^* \underset{kr}{\overset{kf}{\rightleftarrows}} O_2^{*-} + H^+ \quad \text{(A5)}$$

where $kf = 7.14\ x\ 10^5 s^{-1}$, and $kr = 4.78\ x\ 10^{10} M^{-1} s^{-1}$ (Klassen and Ross, 1997).

Simulation results using these rate constants demonstrate that after a short transition period, reaction A5 is close to the equilibrium. Thus, it can be assumed that

$$\frac{[O_2^{*-}][H^+]}{[HO_2^*]} \approx K_A = \frac{kf}{kr} = 1.5\ x\ 10^{-5}\ M \quad \text{(A6)}$$

If the total radicals concentration (C_T) is:

$$C_T = [HO_2^*] + [O_2^{*-}] \quad \text{(A7)}$$

Then, combining A4, A6, A7, and simplifying, the following can be obtained

$$v_{O2} = \left(\frac{[H^+]}{K_A + [H^+]}\right)\left[k_H\left(\frac{[H^+]}{K_A + [H^+]}\right) + k_{OH}\left(\frac{K_A}{K_A + [H^+]}\right)\right] C_T{}^2 \quad \text{(A8)}$$

Finally, from the comparison between A1 with A8

$$k_{obs} = \left(\frac{[H^+]}{K_A + [H^+]}\right)\left[k_H\left(\frac{[H^+]}{K_A + [H^+]}\right) + k_{OH}\left(\frac{K_A}{K_A + [H^+]}\right)\right] \quad \text{(A9)}$$

According to eq.(A9), and using the above-mentioned rate constants, the observed rate constant at pH = 9 is $k_{obs} = 5.05\ x\ 10^3 M^{-1} s^{-1}$. This value was used for all further calculations.

Item 2. From the experimental UV-Vis spectra of the reaction mixture, the concentration of each enzyme species was obtained assuming that the absorbance of the reaction mixture at a given wavelength (A_λ) can be represented as the sum of absorbances corresponding to all the enzymatic species in the mixture:

$$A_\lambda = \varepsilon_{0,\lambda}[E_0] + \varepsilon_{2,\lambda}[E_2] + \varepsilon_{3,\lambda}[E_3] + \varepsilon_{x,\lambda}[E_X] \quad \text{(1)}$$

where $\varepsilon_{i,\lambda}$ represents the extinction coefficient of the enzymatic species i at λ (Fig. 1 of the manuscript). Then, the concentration of each enzyme species was evaluated by fitting eq.(1) to the experimental UV-

Vis spectrum. Figure A1 shows two typical examples of the fitting procedure; Table A1 shows the fitting results.

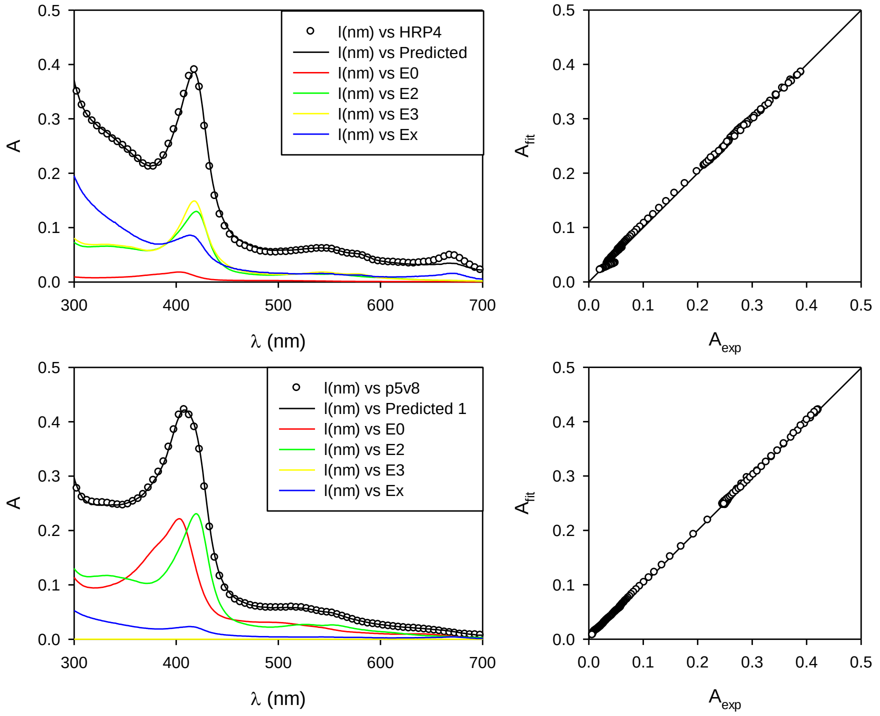

Figure A1. Right panel: typical examples of the fitting procedure. Dots indicate the experimental spectra. Black lines are the fitting results. Color lines indicate the contribution to each species to the total UV-Vis spectrum. Left panel: linear regression between fitted and experimental data.

Table A1. Fitting results of eq.(1) to the UV-Vis spectra showed in Figure A1. All concentrations are expressed in µM.

Sample	E_0	E_2	E_3	E_X	r^2
HRP4	0.18 ± 0.02	1.24 ± 0.12	1.38 ± 0.11	2.80 ± 0.02	0.9985
p5v8	2.15 ± 0.01	2.20 ± 0.05	n.d.	0.76 ± 0.08	0.9999

n.d.: not detectable

Item 3. Figure 3 shows that under the tested conditions (HRP = 5 µM, hydrogen peroxide = 6.5 µM, pH 9), enzyme species E_0 and E_2 represented more than 99% of the total enzyme. Considering that initially HRP was at the resting state (E_0), the initial absorbance at a given wavelength ($A_{i,\lambda}$) is:

$$A_{i,\lambda} = \varepsilon_{0,\lambda} E_T \quad \text{(A10)}$$

However, when hydrogen peroxide is added to the reaction mixture all the enzyme is converted to E_2:

$$A_{t,\lambda} = \varepsilon_{2,\lambda} E_T \quad \text{(A11)}$$

To improve the quality of the results it is desirable to maximize the difference between $A_{i,\lambda}$ and $A_{t,\lambda}$ (ΔA_λ):

$$\Delta A_\lambda = (\varepsilon_{2,\lambda} - \varepsilon_{0,\lambda}) E_T \quad \text{(A12)}$$

Thus, for a given enzyme concentration ΔA_λ only depends on the difference between $\varepsilon_{2,\lambda}$ and $\varepsilon_{0,\lambda}$. Figure A2 shows that this difference reaches a maximum at 422 nm. For this reason, this wavelength was selected to follow the interconversion between E_0 and E_2 as a function of time.

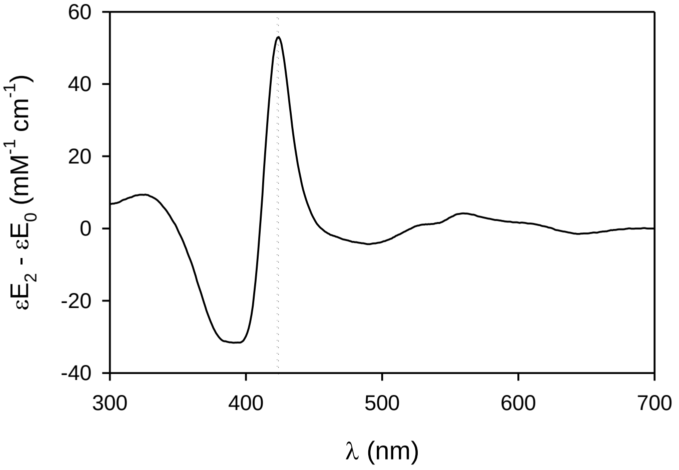

Figure A2. Difference between UV-Vis spectra corresponding to E_2 and E_0. The dotted line indicates the maximum difference at 422 nm.

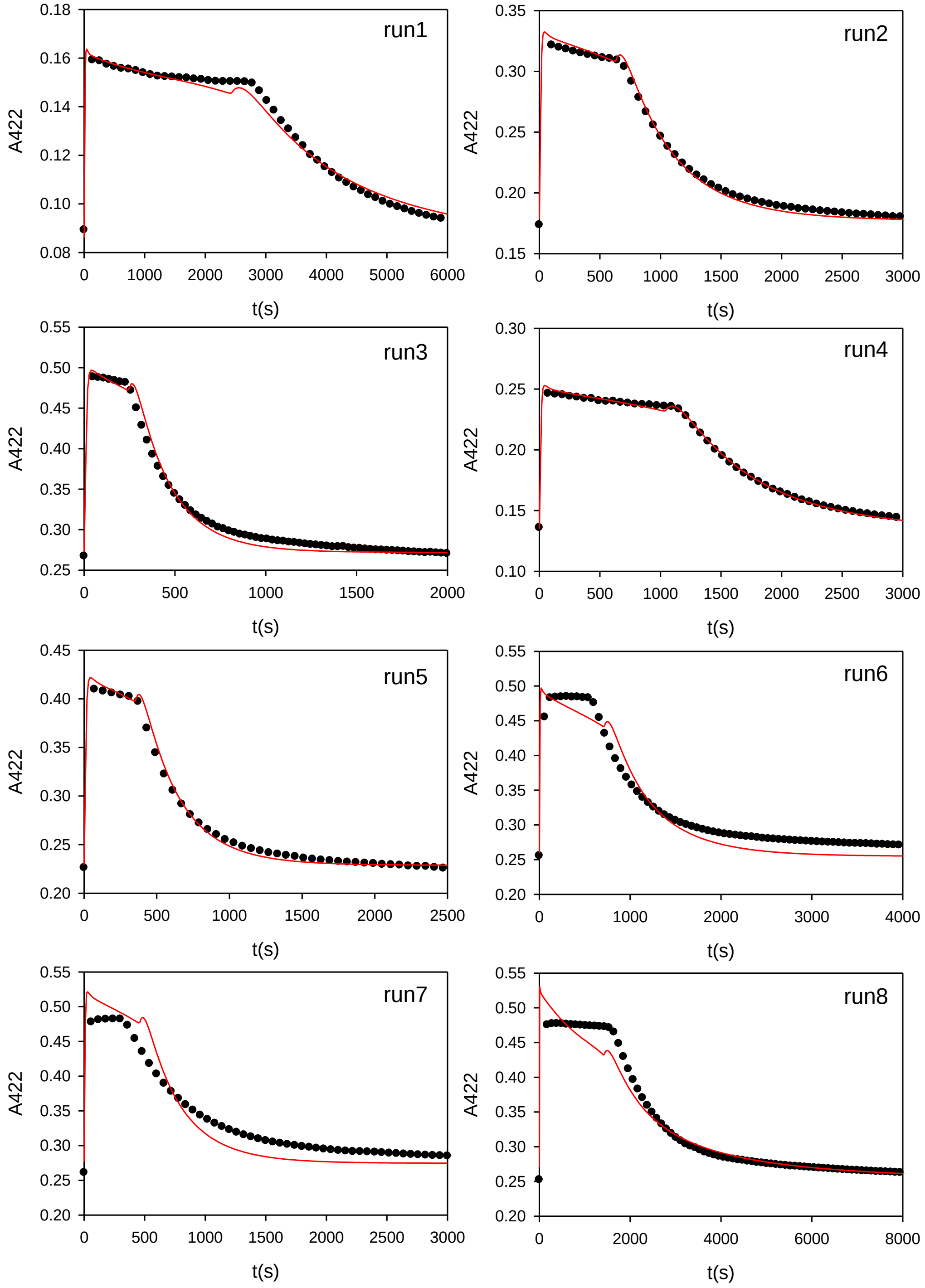

Figure A3. Change of absorbance at 422 nm as a function of time during the decomposition of hydrogen peroxide by HRP (full results). Red lines indicate the proposed model (R1 to R9) using the coefficients shown in Table 1. Initial conditions are shown in Table A2

Table A2. Decomposition of hydrogen peroxide by HRP. Initial conditions corresponding to Figure Ax. All assays were performed in phosphate buffer (100 mM, pH 9) at room temperature

Run	HRP (μM)	H_2O_2 (μM)	Run	HRP (μM)	H_2O_2 (μM)
1	1.7	26	5	4.4	26
2	3.4	26	6	5.0	67
3	5.2	26	7	5.4	48
4	2.7	26	8	5.3	118

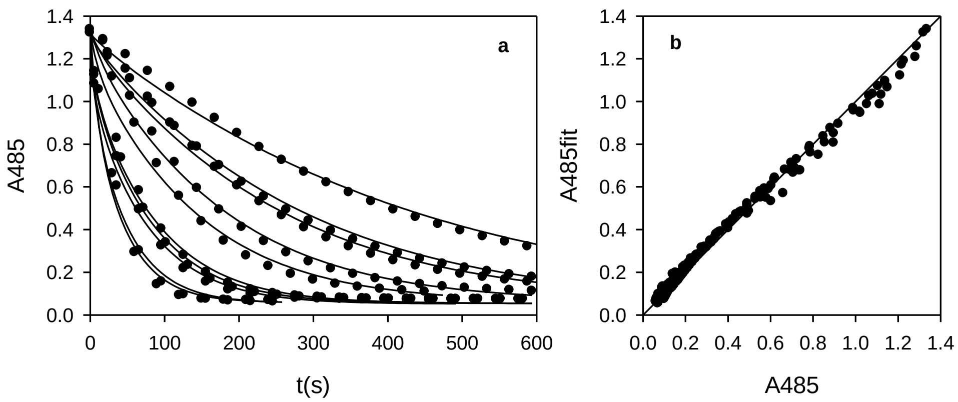

Figure A4. a) Full results corresponding to the peroxidatic activity (decolourization of Orange II) depicted in Figure 6. Lines represent the proposed model (R1 to R9, and eq. 2) using the coefficients shown in Table 1. b) Experimental as a function of calculated absorbance at 485nm. Line represents the perfect correlation (e.g., y = x)

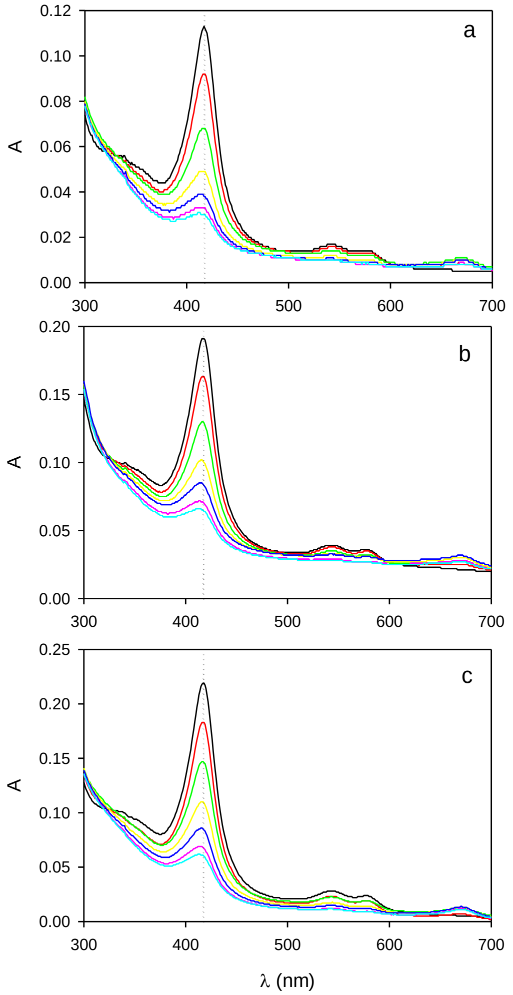

Figure A5. Change of UV/Vis spectra corresponding to different initial concentrations of HRP (in μM): a) 1.0, b) 1.5, c) 2.0. In all cases the initial hydrogen peroxide concentration was 0.0055 M. All measurements were performed in PB (100 mM, pH 9) at room temperature. Dotted lines indicate the peak corresponding to E_3 at 417 nm. The corresponding enzyme species concentrations are shown in Figure 7 of the manuscript.

Item 4. A first set of simulations were performed to assess the effect of the initial external substrate (S_{ei}) and hydrogen peroxide concentrations (H_2O_{2i}) on the initial decolorization rate (VD). Figure 6Aa shows a Haldane-like (e.g., substrate inhibition) dependence of VD (a.u./s) as a function of the initial H_2O_2 concentration:

$$VD = \alpha \frac{H_2O_{2i}}{K_P + H_2O_{2i} + \frac{H_2O_{2i}^2}{K_I}} \quad \text{(A13)}$$

where α (a.u./s), K_P (M), and K_I (M) are the Haldane coefficients. Figure A6 shows that eq.(A13) represented adequately the simulated data. Fitting results (Table A3) demonstrate that coefficients corresponding to eq.(A13) depended on the initial OII concentrations, as it was reported by Morales Urrea et al. (2018).

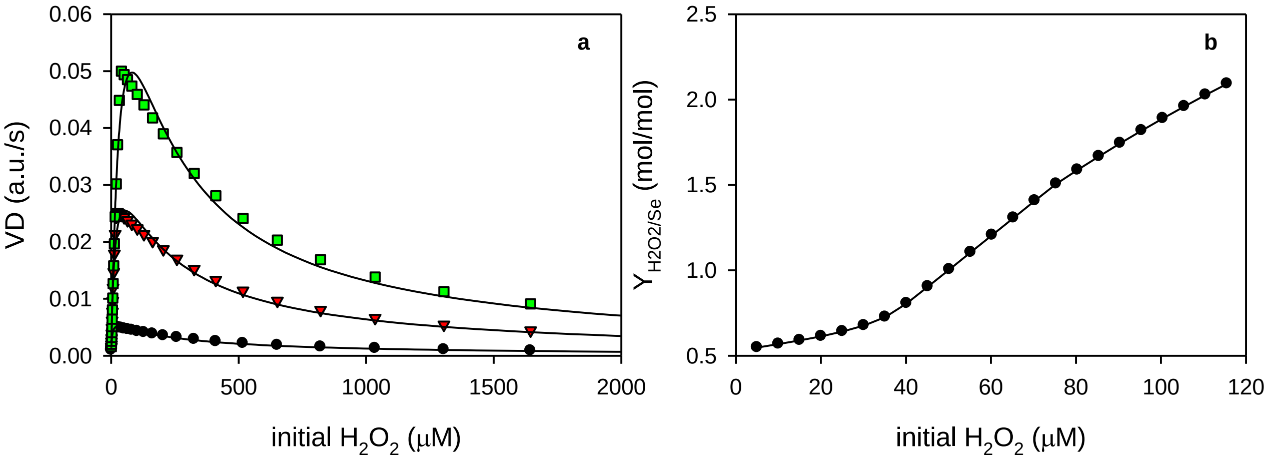

Figure A6. Effect of the initial hydrogen peroxide concentration on a) the observed decolorization rate (VD), and b) the hydrogen peroxide yield ($Y_{H2O2/Se}$, eq. A5). Simulated conditions in a): HRP = 5 μM; S_e = 10 μM (black circles), 50 μM (red triangles), 100 μM (green squares); t = 25 s. In each case, lines represent the fitting results (Table A3) corresponding to the Haldane equation (eq. A13). Simulated conditions in b): HRP = 5 μM; S_e = 50 μM (red triangles); t = 500 s.

Table A3. Fitting results of eq.(A13) to the data shown in Figure A6

**Coefficient**	**OII = 1x10^{-5} M**	**OII = 5x10^{-5} M**	**OII = 1x10^{-4} M**
α (a.u./s)	0.0064 ± 0.0001	0.041 ± 0.001	0.109 ± 0.004
K_P (M)	(3.6 ± 0.2)x10^{-6}	(1.7 ± 0.1)x10^{-5}	(4.9 ± 0.3)x10^{-5}
K_I (M)	(2.4 ± 0.1)x10^{-4}	(1.9 ± 0.1)x10^{-4}	(1.4 ± 0.1)x10^{-4}

A second set of simulations were performed to assess the effect of the initial hydrogen peroxide concentration (H_2O_{2i}) on the amount of consumed H_2O_2 per mol of oxidized external substrate (OII) ($Y_{H2O2/Se}$). In these simulations, the initial HRP and OII concentrations were 5 μM, and 50 μM, respectively. Then, the hydrogen peroxide yield ($Y_{H2O2/Se}$) was obtained as follows:

$$Y_{H2O2/Se} = \frac{H2O2_i - H2O2_t}{Se_i - Se_t} \quad \text{(A14)}$$

where the subscripts i and t indicate the initial condition and the respective value at a given time t. Results depicted in Figure 6Ab demonstrate that $Y_{H2O2/Se}$ increase as a function of the initial H_2O_2 concentration, which is in agreement with the results reported by Morales Urrea et al. (2018).

Item 5. A pseudo-steady state approximation of the proposed model was used to calculate the fraction of each enzyme species. Because species E_x (R7 in the manuscript) is not active, we focused attention on active species E_0, E_1, E_2, and E_3. Accordingly, the active enzyme concentration (E_a) is

$$E_a = [E_0] + [E_1] + [E_2] + [E_3] \quad \text{(A15)}$$

Considering that species E_0, E_1, and E_2 are close to a pseudo-steady state, the following expressions can be obtained:

$$\frac{d[E_0]}{dt} = -k_o[E_0][P] + (k_{2s}[S] + k_{2si}[S_i])[E_2] + k_3[E_3] \cong 0 \quad \text{(A16)}$$

$$\frac{d[E_1]}{dt} = k_o[E_0][P] - \left(k_{1s}[S] + k_{1si}[S_i] + k_{1p}[P]\right)[E_1] \cong 0 \quad \text{(A17)}$$

$$\frac{d[E_2]}{dt} = \left(k_{1s}[S] + k_{1si}[S_i] + k_{1p}[P]\right)[E_1] - \left(k_{2s}[S] + k_{2si}[S_i] + k_{2p}[P]\right)[E_2] \cong 0 \quad \text{(A18)}$$

where the symbol P was used instead of H_2O_2 to simplify the notation.

Reordering eqs. (A17) and (A18):

$$\frac{[E_1]}{[E_0]} = \frac{k_o[P]}{k_{1s}[S]+k_{1si}[S_i]+k_{1p}[P]} \quad \text{(A19)}$$

$$\frac{[E_2]}{[E_1]} = \frac{k_{1s}[S]+k_{1si}[S_i]+k_{1p}[P]}{k_{2s}[S]+k_{2si}[S_i]+k_{2p}[P]} \quad \text{(A20)}$$

Then, the combination of eqs. (A19) and (A20) yields the following:

$$\frac{[E_2]}{[E_0]} = \frac{k_o[P]}{k_{2s}[S]+k_{2si}[S_i]+k_{2p}[P]} \quad \text{(A21)}$$

Finally, using (A16):

$$(k_{2s}[S] + k_{2si}[S_i])\frac{[E_2]}{[E_0]} + k_3\frac{[E_3]}{[E_0]} = k_o[P] \quad \text{(A22)}$$

Combining (A21) with (A22) and simplifying:

$$\frac{[E_3]}{[E_0]} = \frac{k_o}{k_3}[P]\left(\frac{k_{2p}[P]}{k_{2s}[S]+k_{2si}[S_i]+k_{2p}[P]}\right) \quad \text{(A23)}$$

Then, reordering (A15) and combinig with eqs.(A19), (A21), and (A23):

$$\frac{Ea}{[E_0]} = 1 + \frac{k_o[P]}{k_{1s}[S]+k_{1si}[S_i]+k_{1p}[P]} + \frac{k_o[P]}{k_{2s}[S]+k_{2si}[S_i]+k_{2p}[P]} + \frac{k_o}{k_3}[P]\left(\frac{k_{2p}[P]}{k_{2s}[S]+k_{2si}[S_i]+k_{2p}[P]}\right) \quad \text{(A24)}$$

$$\frac{Ea}{[E_0]} = \frac{(k_{1s}[S]+k_{1si}[S_i]+k_{1p}[P])(k_{2s}[S]+k_{2si}[S_i]+k_{2p}[P])+k_o[P](k_{2s}[S]+k_{2si}[S_i]+k_{2p}[P])+k_o[P](k_{1s}[S]+k_{1si}[S_i]+k_{1p}[P])+\frac{k_ok_{2p}}{k_3}[P]^2(k_{1s}[S]+k_{1si}[S_i]+k_{1p}[P])}{(k_{1s}[S]+k_{1si}[S_i]+k_{1p}[P])(k_{2s}[S]+k_{2si}[S_i]+k_{2p}[P])}$$

Defining

$$D = (k_{1s}[S] + k_{1si}[S_i] + k_{1p}[P])(k_{2s}[S] + k_{2si}[S_i] + k_{2p}[P]) + k_o[P](k_{2s}[S] + k_{2si}[S_i] + k_{2p}[P]) + k_o[P](k_{1s}[S] + k_{1si}[S_i] + k_{1p}[P]) + +\frac{k_ok_{2p}}{k_3}[P]^2(k_{1s}[S] + k_{1si}[S_i] + k_{1p}[P]) \quad \text{(A25)}$$

Using (A25), the fraction of each species as a function of S, Si, and P can be obtained as follows:

$$f_0 = \frac{[E_0]}{Ea} = \frac{(k_{1s}[S]+k_{1si}[S_i]+k_{1p}[P])(k_{2s}[S]+k_{2si}[S_i]+k_{2p}[P])}{D} \quad \text{(A26)}$$

$$f_1 = \frac{[E_1]}{Ea} = \frac{k_o[P](k_{2s}[S]+k_{2si}[S_i]+k_{2p}[P])}{D} \quad \text{(A27)}$$

$$f_3 = \frac{[E_2]}{Ea} = \frac{k_o[P](k_{1s}[S]+k_{1si}[S_i]+k_{1p}[P])}{D} \quad \text{(A28)}$$

$$f_3 = \frac{[E_3]}{Ea} = \frac{\frac{k_o k_{2p}}{k_3}[P]^2(k_{1s}[S]+k_{1si}[S_i]+k_{1p}[P])}{D} \quad \text{(A29)}$$

Equations (A26) to (A29) along with the coefficients shown in Table 1 were used to calculate the fraction of each species as a function of S, Si, and P (Fig. 9 of the manuscript).

According to the proposed model, the consumption rate of the oxidant (R_P) is given by reactions R1, R4, and R5:

$$R_P = (k_0 E_0 + k_{1p} E_1 + k_{2p} E_2)[P] \quad \text{(A30)}$$

Then, combining eqs.(A26) to (A28) in (A30)

$$R_P = (k_0 f_0 + k_{1p} f_1 + k_{2p} f_2)[P]E_a \quad \text{(A31)}$$

Figure A7 demonstrates that the presence of an external substrate (S_e) enhances the consumption rate of the oxidant (R_P). In particular, at low hydrogen peroxide concentrations R_P in the absence of S_e (black line in Fig. A7) is about 2 order of magnitude lower than R_P in the presence of the substrate (red line in Fig. A7). This difference can be mainly attributted to the difference in the values corresponding to f_0 in the presence and the absence of S_e (see Fig. 9 in the manuscript).

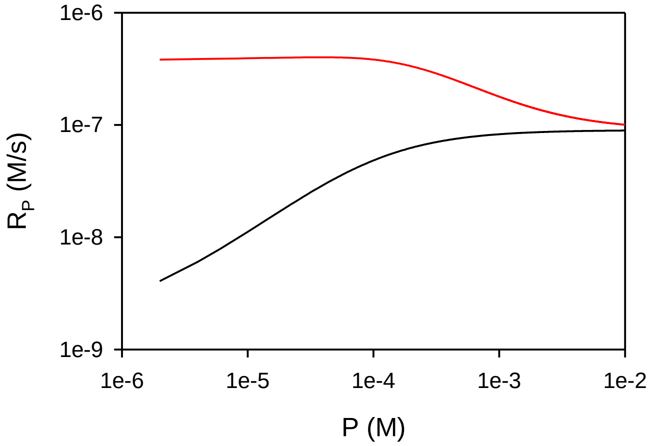

Figure A7. Effect of the hydrogen peroxide concentration (P) on the the consumption rate of the oxidant (R_P) under the presence (S_e = 1x10^{-4}M, red line) and the absence (S_e = 0, black line) of an external substrate (S_e). In both cases E_a = 1x10^{-6}M. Equations (A26) to (A31) along with the coefficients shown in Table 1 were used in all calculations.

References

Morales Urrea D.A., Haure P.M., García Einschlag F.S., Contreras E.M. (2018a) Horseradish peroxidase-mediated decolourization of Orange II: modelling hydrogen peroxide utilization efficiency at different pH values. Environ. Sci. Pollut. Res. 25(20):19989-20002.

Klassen N.V., Ross C.K. (1997). Water calorimetry: the heat defect. J. Res. Natl. Inst. Stand. Technol. 102, 63-74.
